# Modification of acetoacetyl-CoA reduction step in *Ralstonia eutropha* for biosynthesis of poly(3-hydroxybutyrate-*co*-3-hydroxyhexanoate) from structurally unrelated compounds

**DOI:** 10.1186/s12934-019-1197-7

**Published:** 2019-08-29

**Authors:** Mengxiao Zhang, Shunsuke Kurita, Izumi Orita, Satoshi Nakamura, Toshiaki Fukui

**Affiliations:** School of Life Science and Technology, Tokyo Institute of Technology, 4259 Nagatsuta, Midori-ku, Yokohama, 226-8501 Japan

## Abstract

**Background:**

Poly((*R*)-3-hydroxybutyrate-*co*-(*R*)-3-hydroxyhexanoate) [P(3HB-*co*-3HHx)] is a bacterial polyester with high biodegradability, even in marine environments. *Ralstonia eutropha* has been engineered for the biosynthesis of P(3HB-*co*-3HHx) from vegetable oils, but its production from structurally unrelated carbon sources remains unsatisfactory.

**Results:**

*Ralstonia eutropha* strains capable of synthesizing P(3HB-*co*-3HHx) from not only fructose but also glucose and glycerol were constructed by integrating previously established engineering strategies. Further modifications were made at the acetoacetyl-CoA reduction step determining flux distribution responsible for the copolymer composition. When the major acetoacetyl-CoA reductase (PhaB1) was replaced by a low-activity paralog (PhaB2) or enzymes for reverse β-oxidation, copolyesters with high 3HHx composition were efficiently synthesized from glucose, possibly due to enhanced formation of butyryl-CoA from acetoacetyl-CoA via (*S*)-3HB-CoA. P(3HB-*co*-3HHx) composed of 7.0 mol% and 12.1 mol% 3HHx fractions, adequate for practical applications, were produced at cellular contents of 71.4 wt% and 75.3 wt%, respectively. The replacement by low-affinity mutants of PhaB1 had little impact on the PHA biosynthesis on glucose, but slightly affected those on fructose, suggesting altered metabolic regulation depending on the sugar-transport machinery. PhaB1 mostly acted in the conversion of acetoacetyl-CoA when the cells were grown on glycerol, as copolyester biosynthesis was severely impaired by the lack of *phaB1*.

**Conclusions:**

The present results indicate the importance of flux distribution at the acetoacetyl-CoA node in *R. eutropha* for the biosynthesis of the PHA copolyesters with regulated composition from structurally unrelated compounds.

## Background

Bio-based plastics and biodegradable plastics have attracted much attention as eco-friendly alternatives to petroleum-based plastics. In particular, biodegradable plastics have become increasingly important as recent studies have provided evidence of serious pollution in marine environments due to the debris of synthetic polymers, called microplastics [1, 2]. Polyhydroxyalkanoates (PHAs) are biopolyesters synthesized by a variety of bacteria and some archaea from renewable carbon resources [3, 4]. They are synthesized as an intracellular storage of carbon and energy, generally when cell growth is limited by the depletion of nutrients such as nitrogen, phosphorus, or oxygen in the presence of excess carbon sources. It has been demonstrated that PHAs show high biodegradability in various environments, including marine ones [5–7].

Poly[(*R*)-3-hydroxybutyrate] [P(3HB)], a homopolyester of (*R*)-3-hydroxybutyrate, is the most abundant PHA in nature. In many P(3HB)-producing bacteria, two molecules of acetyl-CoA are converted to (*R*)-3-hydroxybutyryl (3HB)-CoA by β-ketothiolase (PhaA) and NADPH-dependent acetoacetyl-CoA reductase (PhaB) and then polymerized to P(3HB) by the function of PHA synthase (PhaC). Unfortunately, the range of application of P(3HB) has been limited due to its high crystallinity and brittleness. Substantial research has therefore been conducted on the microbial synthesis of PHA copolymers exhibiting better mechanical properties by adding precursor compounds into the cultivation medium, as well as metabolic engineering [4]. Poly((*R*)-3-hydroxybutyrate-*co*-(*R*)-3-hydroxyhexanoate) [P(3HB-*co*-3HHx)], a kind of short-chain-length/medium-chain-length-PHA copolymer, is one of the most practical PHAs because the copolyester composed of > 10 mol% of the C_6_ fraction shows more flexible properties with lower melting temperature and crystallinity than P(3HB) homopolymer [8]. This copolyester was initially identified as a PHA synthesized by *Aeromonas caviae* from vegetable oils and fatty acids [8, 9]. The biosynthesis genes in *A. caviae* are clustered as *phaP*-*C*-*J*_*Ac*_ encoding PHA granule-associated protein (phasin), PHA synthase with unique substrate specificity to 3HA-CoAs of C_4_ to C_7_, and short-chain-length-specific (*R*)-specific enoyl-CoA hydratase (*R*-hydratase), respectively [10–12].

*Ralstonia eutropha* (*Cupriavidus necator*) strain H16 is known as an efficient producer of P(3HB) from fructose, gluconate, vegetable oils, and fatty acids. The P(3HB) biosynthesis genes are clustered on chromosome 1 as *phaC*-*A*-*B1* [13]. PhaB1 is the major acetoacetyl-CoA reductase for the supply of (*R*)-3HB-CoA for polymerization, while the weakly expressed paralog PhaB3 partially supports P(3HB) synthesis in *phaB1*-deleted strains grown on fructose [14]. BktB, a β-ketothiolase paralog having broader substrate specificity than PhaA, is involved in the generation of C_5_ intermediates for the biosynthesis of poly((*R*)-3-hydroxybutyrate-*co*-(*R*)-3-hydroxyvalerate) copolymer with propionate supplementation [15]. Several studies have focused on the metabolic engineering of *R. eutropha* for the biosynthesis of P(3HB-*co*-3HHx) from vegetable oils [16–21]. Meanwhile, considering the inexpensiveness and availability of sugars and glycerol, P(3HB-*co*-3HHx) production from such structurally unrelated compounds is an important technology to be established. This is also an interesting challenge in metabolic engineering because no wild microbes capable of synthesizing P(3HB-*co*-3HHx) from sugars have been known so far. We have therefore focused on this issue and achieved the biosynthesis of P(3HB-*co*-22 mol% 3HHx) from fructose by an engineered strain of *R. eutropha* equipped with an artificial pathway [22, 23]. The key step in this pathway is the formation of butyryl-CoA from crotonyl-CoA by the combination of crotonyl-CoA carboxylase/reductase (Ccr) [24] and ethylmalonyl-CoA decaroboxylase (Emd) [25], through which ethylmalonyl-CoA formed from crotonyl-CoA by reductive carboxylase activity of Ccr was converted back to butyryl-CoA by Emd. Butyryl-CoA was then condensed with acetyl-CoA to form 3-oxohexanoyl-CoA and further converted to (*R*)-3HHx-CoA, which was copolymerized with (*R*)-3HB-CoA by PhaC_NSDG_, a N149S/D171G mutant of PHA synthase from *A. caviae* [26]. One drawback of *R. eutropha* H16 is the rather narrow spectrum of utilizable carbon sources, as this strain is unable to utilize glucose, xylose, and arabinose and grows slowly on glycerol. The substrate utilization range of *R. eutropha* H16 has been expanded by genetic modification [27]. For examples, the strains capable of utilizing glucose [28–30], mannose [30], sucrose [31], as well as that with enhanced glycerol utilization [32] have been reported.

In the previous P(3HB-*co*-3HHx) biosynthesis from fructose by the artificial pathway in *R. eutropha*, we observed that the deletion of *phaB1* was an important modification to achieve a high 3HHx fraction in the copolyester, but this was accompanied by a decrease of PHA production [23]. In this study, we modified the acetoacetyl-CoA reduction step in *R. eutropha* to establish enough flux for the formation of both (*R*)-3HB-CoA and (*R*)-3HHx-CoA from acetoacetyl-CoA, aiming at the efficient biosynthesis of P(3HB-*co*-3HHx) composed of ~ 10 mol% of the C_6_ unit, potentially suitable for practical applications, from structurally unrelated carbon sources.

## Results

### Integrated engineering of *R. eutropha* for P(3HB-*co*-3HHx) biosynthesis from glucose and glycerol

*Ralstonia eutropha* strain NSDG was previously constructed by replacing the original PHA synthase gene (*phaC*) on chromosome 1 with *phaC*_NSDG_ encoding a mutant of PHA synthase derived from *A. caviae* [33]. It has been demonstrated that PhaC_NSDG_ can synthesize P(3HB-*co*-3HHx) with higher levels of 3HHx than the wild-type enzyme [26]. A recombinant strain of *R. eutropha* capable of synthesizing the copolyester from glucose and glycerol was constructed based on the strain NSDG by the integration of three further engineering strategies. Glucose-assimilation ability was conferred by the disruption of *nagR* and mutation in *nagE* corresponding to substitution of Gly265 by Arg in the EIIC-EIIB component of the GlcNAc-specific phosphoenolpyruvate sugar phosphotransferase (PTS) system (Additional file 1: Fig. S1A) [28]. *glpFK* genes derived from *E. coli* were then inserted into chromosome 1 in order to enhance glycerol assimilation (Additional file 1: Fig. S1B) [32]. The resulting strain NSDG-GG highly accumulated P(3HB) homopolymer from glucose, fructose, and glycerol (81.2–83.1 wt% of the dry cell mass) as shown in entries 1, 14, and 27 in Figs. 2, 3 and 4, respectively. The detailed results of the cultivation experiments in this study are shown in Additional file 1: Tables S1–S3. The enhanced P(3HB) production from glucose by this strain has also been reported by Biglari et al. [34]. pBPP-ccr_Me_J4a-emd is a previously constructed plasmid for the biosynthesis of P(3HB-*co*-3HHx) from fructose by *R. eutropha* [23], in which *phaJ4a* derived from *R. eutropha* encodes *R*-hydratase showing higher activity to 2-hexenoyl-CoA than crotonyl-CoA [C_6_ > C_4_] [18]. The introduction of this plasmid into the strain NSDG-GG enabled P(3HB-*co*-3HHx) biosynthesis from glucose and glycerol as well as from fructose with high cellular content (75.2–82.0 wt%), although the 3HHx fractions were 2.3 mol% or less (entries 2, 15, and 28). When the plasmid-borne *phaJ4a* was replaced by *phaJ*_*Ac*_ derived from *A. caviae* [12], encoding the *R*-hydratase specific to short-chain-length-substrate [C_6_ < C_4_], the PHA production tended to decrease (66.2–76.1 wt%) with still low levels of 3HHx fraction (entries 8, 21, and 34).

### Deletion of *phaB1* and the effects on PHA biosynthesis from glucose

**Fig. 1:**
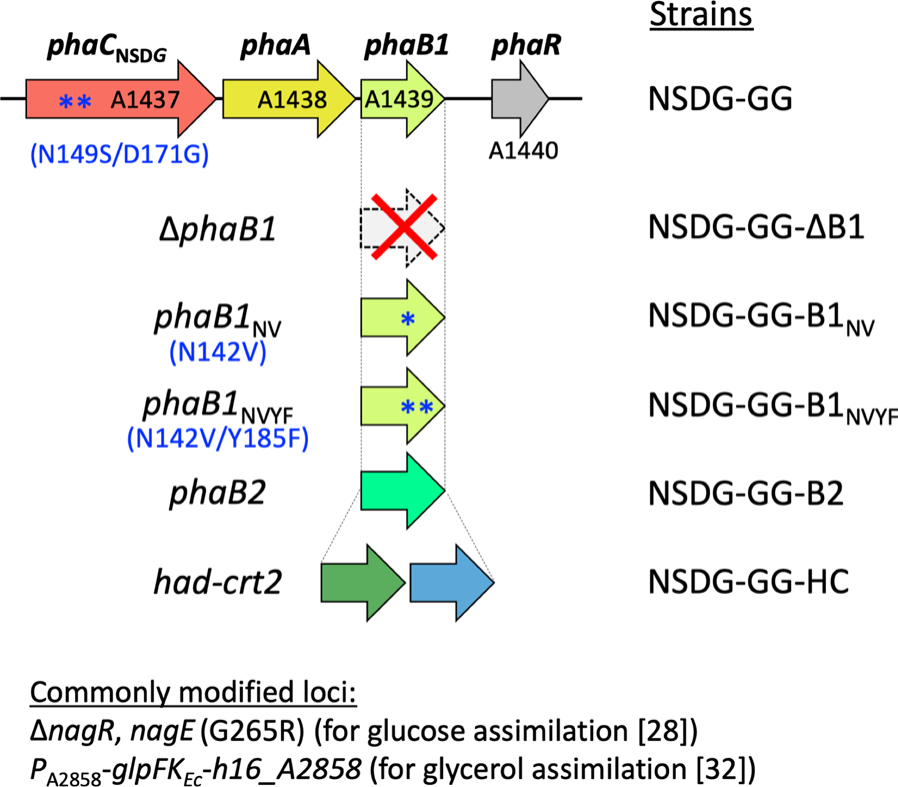
Schematic diagram of genotypes of *R. eutropha* NSDG-GG-based recombinant strains. *phaC*_NSDG_, a gene encoding N149S/D171G mutant of PHA synthase from *A. caviae*; *phaA*, β-ketothiolase gene; *phaB1*, a gene encoding major NADPH-acetoacetyl-CoA reductase; *phaR*_*Re*_, PHA-binding transcriptional repressor gene; *phaB1*_NV_ and *phaB1*_NVYF_, genes encoding N142V and N142V/Y185F mutants of PhaB1; *phaB2*, a gene encoding minor NADPH-acetoacetyl-CoA reductase; *had*, NAD-(*S*)-3HB-CoA dehydrogenase gene, *crt2*, crotonase gene. The commonly modified loci in these strains are shown in Additional file 1: Fig. S1

**Fig. 2:**
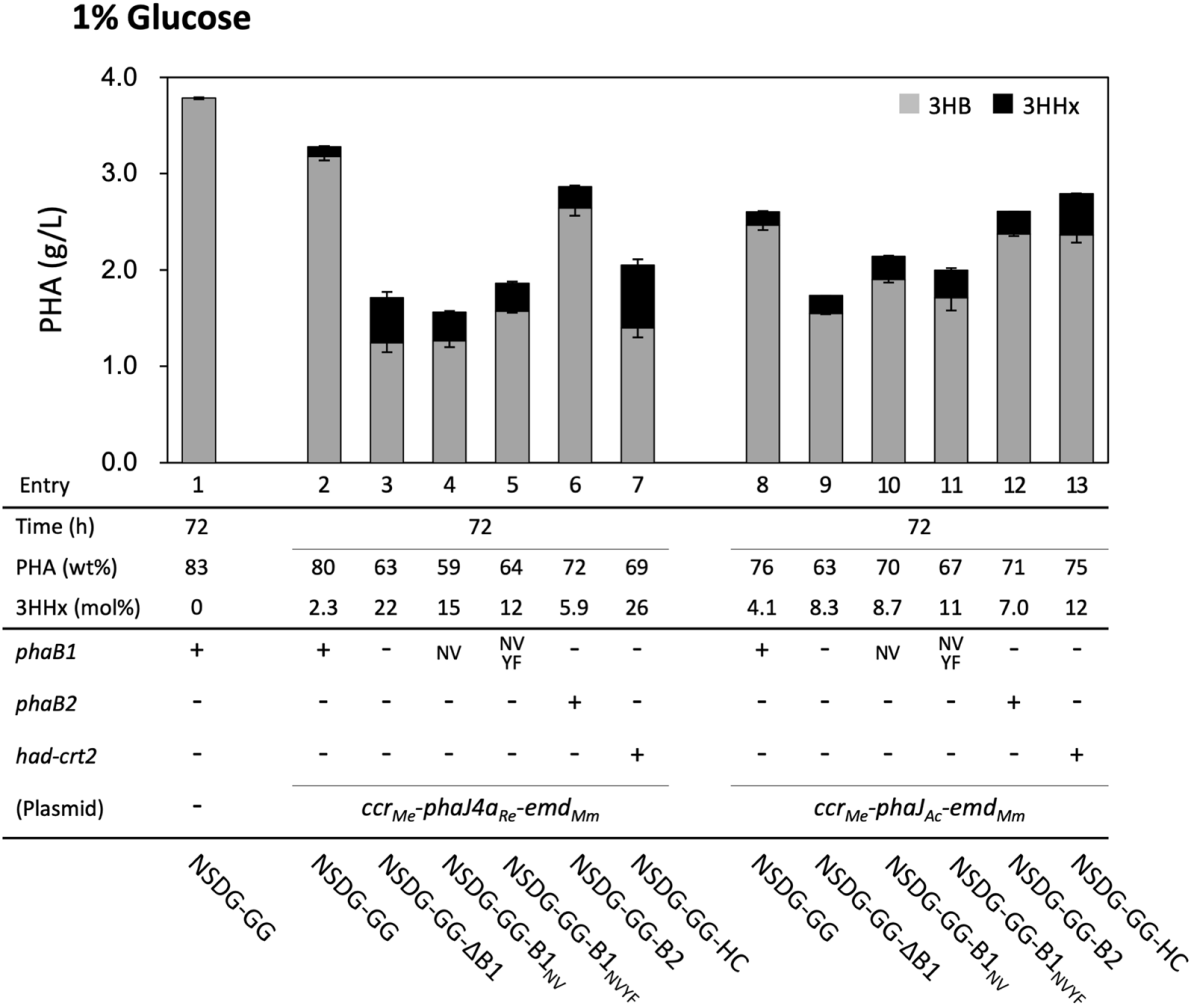
P(3HB-*co*-3HHx) biosynthesis by NSDG-GG-based engineered strains of *R. eutropha* from glucose. The amounts of 3HB and 3HHx units in PHA are shown in gray and black bars, respectively. The cells were cultivated in a 100 ml MB medium containing 1% (w/v) glucose for 72 h at 30 °C (n = 3)

The *phaB1*-deleted strain NSDG-GG-∆B1 (Fig. 1) harboring pBPP-ccr_Me_J4a-emd produced the copolyester composed of a much higher 3HHx fraction (22.0 mol%) with lower content (62.5 wt%) from glucose (entry 3 in Fig. 2) when compared with the corresponding *phaB1*^+^ strain (entry 2). Such effects of *phaB1*-deletion were also observed for the strains harboring PhaJ_*Ac*_ (pBPP-ccr_Me_J_Ac_-emd) [entries 9 (∆*phaB1*) and 8 (*phaB1*^+^)], although the increase in 3HHx composition was less significant (up to 8.3 mol%) than that of the strain having PhaJ4a. These compositional changes caused by the *phaB1* deletion, also seen in the previous study [23], were due to not only a relative increase in 3HHx units attributed to a decrease in 3HB units, but also a net increase in C_6_ units (Additional file 1: Table S1). It was likely that (*R*)-3HB-CoA formation from acetoacetyl-CoA was dominated by the function of PhaB1 showing high catalytic efficiency and being highly expressed [35, 36], while the formation of butyryl-CoA from acetoacetyl-CoA via (*S*)-3HB-CoA became significant when the competing (*R*)-specific reduction was mediated by only PhaB3 and consequently weakened in the absence of PhaB1. We then investigated the effects of introducing low-activity mutants or paralog of PhaB1, or enzymes for reverse β-oxidation, on the copolyester biosynthesis properties, as described below.

### Introduction of low affinity mutants of PhaB1 and the effects on PHA biosynthesis from glucose

We had attempted protein engineering of PhaB1 based on the crystal structure [37], and obtained mutants with low affinity toward acetoacetyl-CoA, as described in Additional Information. The kinetic parameters of PhaB1 and the mutants using *N*-His_6_-tagged recombinant proteins are shown in Table 1. We confirmed very high affinity of PhaB1 to acetoacetyl-CoA with a *K*_*m*_ value of 2 μM, as previously reported [37, 38], and observed substrate inhibition at acetoacetyl-CoA concentrations higher than 12 μM. N142V and Y185F mutants of PhaB1 (designated as PhaB1_NV_ and PhaB1_YF_) showed much larger *K*_m_ values toward acetoacetyl-CoA, whereas *V*_*max*_ values were not affected by the N142V mutation and retained at 78% by the Y185F mutation, when compared with those of the parent wild-type enzyme. The double mutations of N142V and Y185F markedly decreased catalytic efficiency, as *K*_*m*_ and *V*_*max*_ of the double mutant PhaB1_NVYF_ to acetoacetyl-CoA increased 50-fold and decreased by one-third of those of PhaB1, respectively.

**Table 1:** Kinetic parameters of PhaB paralogs and PhaB1 mutants from *R. eutropha* toward acetoacetyl-CoA

Enzyme	Mutation(s)	*K*_*m*_ [μM]	*V*_*max*_ [U mg^−1^]	*k*_cat_ [s^−1^]	*k*_cat_/*K*_*m*_ [μM^−1^ s^−1^]
PhaB1		1.99 ± 0.23	162 ± 6	71.2 ± 2.7	35.8 ± 4.4
PhaB1_NV_	N142V	58.5 ± 18.1	175 ± 31	76.7 ± 2.7	1.31 ± 0.47
PhaB1_YF_	Y185F	86.2 ± 20.6	127 ± 17	55.8 ± 7.6	0.65 ± 0.18
PhaB1_NVYF_	N142V/Y185F	109 ± 38	53.9 ± 8.3	23.7 ± 3.7	0.22 ± 0.08
PhaB2		2.48 ± 0.63	10.5 ± 0.6	4.90 ± 0.28	2.00 ± 0.52
PhaB3		1.27 ± 0.53	88.9 ± 8.7	40.8 ± 4.0	32.7 ± 14.0

PhaB1_NV_ (low-affinity) and PhaB1_NVYF_ (low-affinity and low-activity) were here applied with the aim of achieving moderate weakening of the (*R*)-specific reduction step in the P(3HB-*co*-3HHx) biosynthesis pathway. The two mutant genes were individually introduced into the strain NSDG-GG-∆B1 downstream of *phaA* in the chromosome of NSDG-GG-∆B1 (original *phaB1* locus) (Fig. 1), and the resulting strains NSDG-GG-B1_NV_ and NSDG-GG-B1_NVYF_ were used as hosts for pBPP-ccr_Me_J4a-emd or pBPP-ccr_Me_J_Ac_-emd. When these strains were cultivated on glucose, however, unexpectedly the copolyester biosynthesis properties (entries 4, 5, and 11 in Fig. 2 and Additional file 1: Table S1) were not greatly changed when compared with those of the *phaB1*-deleted strains (entries 3 and 9), except for NSDG-GG-B1_NV_/pBPP-ccr_Me_J_Ac_-emd (entry 10) that accumulated slightly more P(3HB-*co*-3HHx) than the *phaB1*-deleted strain.

### Introduction of low-activity paralog PhaB2 and the effects on PHA biosynthesis from glucose

Although a previous study revealed the roles of the three PhaB paralogs in P(3HB) biosynthesis by *R. eutropha* [14], the catalytic properties of PhaB2 and PhaB3 had yet to be determined. Table 1 also shows the results of kinetic analysis of PhaB2 and PhaB3 using the *N*-terminal His_6_-tagged recombinant proteins. Both PhaB2 and PhaB3 showed very high affinity to acetoacetyl-CoA, with *K*_*m*_ values of 2.5 μM and 1.3 μM, respectively, and substrate inhibition as well as PhaB1, while the *V*_*max*_ values of PhaB2 and PhaB3 were one order of magnitude lower and about half, respectively, when compared with that of PhaB1.

As PhaB2 was supposed to be applicable as a low-activity reductase for the moderate weakening of the (*R*)-specific reduction of acetoacetyl-CoA, the strain NSDG-GG-B2 was constructed by inserting *phaB2* downstream of *phaA* in the chromosome of NSDG-GG-∆B1 (Fig. 1). The strain transformed with pBPP-ccr_Me_J4a-emd or pBPP-ccr_Me_J_Ac_-emd accumulated P(3HB-*co*-5.9–7.0 mol% 3HHx) with 71.4–72.3 wt% cellular content on glucose (entries 6 and 12 in Fig. 2), which demonstrated that the insertion of *phaB2* into the *pha* operon increased PHA production when compared with the *phaB1*-deleted strains. The amounts of the 3HB and 3HHx units incorporated into the polyester were intermediate between those by NSDG-GG (*phaB1*^+^) and NADG-GG-∆B1 (*phaB1*^–^) strains (Additional file 1: Table S1). This was consistent with the altered flux distribution from acetoacetyl-CoA to (*R*)-3HB-CoA and (*S*)-3HB-CoA by PhaB2. No significant difference in the PHA production was observed between the strains harboring PhaJ_*Ac*_ and PhaJ4a.

### Enhancement of reverse β-oxidation and the effects on PHA biosynthesis from glucose

We recently identified two NAD(H)-dependent (*S*)-3-hydroxyacyl-CoA dehydrogenases, PaaH1 (H16_A0282) and Had (H16_A0602), as well as (*S*)-specific enoyl-CoA hydratase (crotonase) Crt2 (H16_A3307) in the cell extract of *R. eutropha* [39]. Analysis of the enzymatic characteristics indicated that these enzymes showed rather broad specificities with high activities toward the C_4_–C_8_ substrates. We thus utilized Had and Crt2 for P(3HB-*co*-3HHx) biosynthesis, because these enzymes along with endogenous β-ketothiolases (PhaA, BktB, etc.) and Ccr_*Me*_-Emd_*Mm*_ could potentially establish reverse β-oxidation converting three acetyl-CoA molecules to 2-hexenoyl-CoA via (*S*)-3HA-CoA intermediates. A tandem of *had* and *crt2* was inserted downstream of *phaA* in NSDG-GG-∆B1 (Fig. 1), and the resulting strain NSDG-GG-HC was further transformed with the plasmids for the copolyester synthesis. The strains harboring pBPP-ccr_Me_J4a-emd or pBPP-ccr_Me_J_Ac_-emd produced copolyesters with 3HHx compositions of 26.0 mol% and 12.1 mol% from glucose (entries 7 and 13 in Fig. 2), respectively, which were higher than those by the corresponding ∆*phaB1* strains (entries 3 and 9). Although the increase of 3HHx composition with PhaJ_*Ac*_ was smaller than that with PhaJ4a, the cellular content of P(3HB-*co*-3HHx) by the strain harboring *phaJ*_*Ac*_ reached up to 75.3 wt%, which was comparable to that of the corresponding *phaB1*^+^ strain NSDG-GG/pBPP-ccr_Me_J_Ac_-emd (entry 8).

### PHA biosynthesis by the engineered *R. eutropha* strains from fructose

**Fig. 3:**
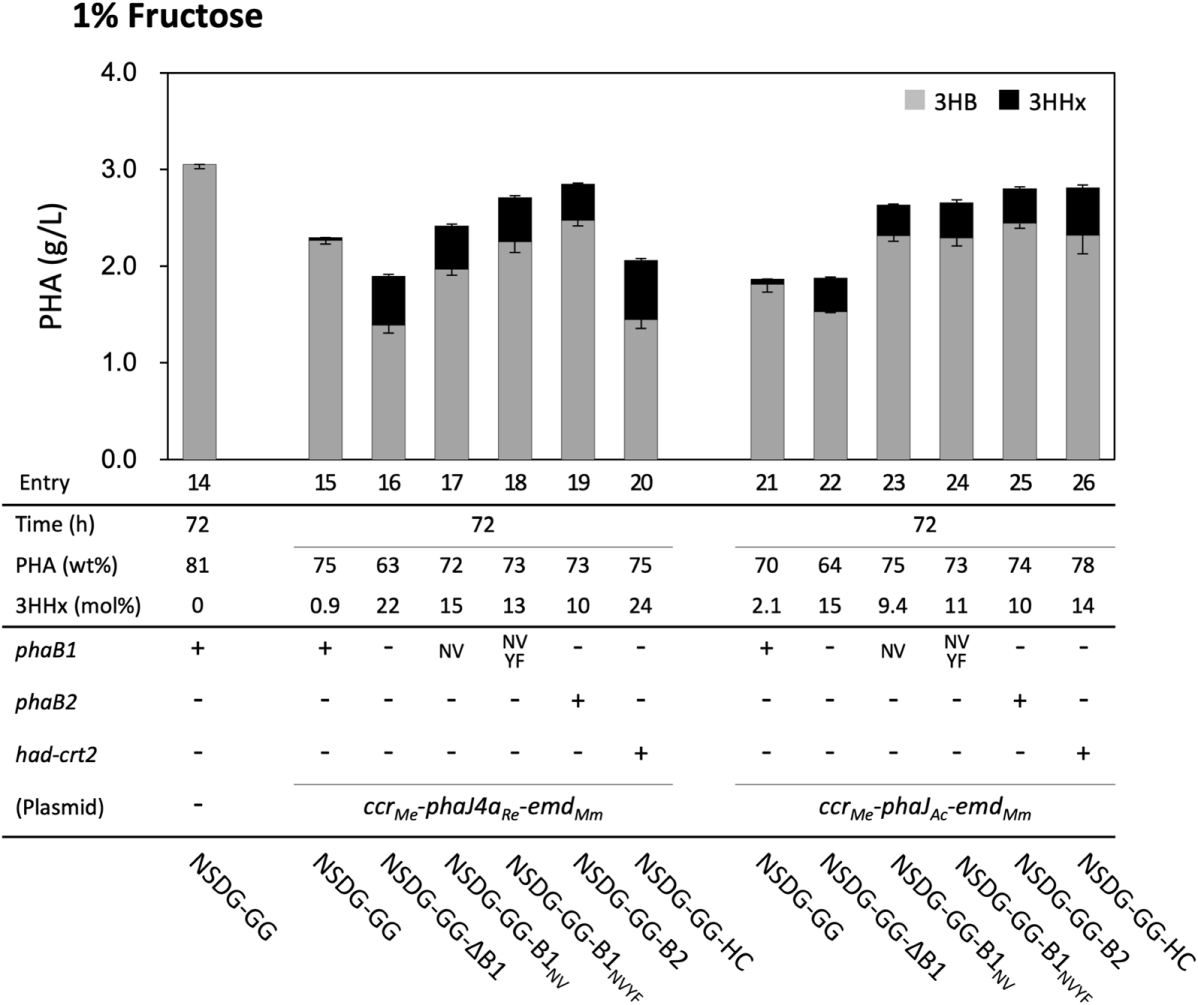
P(3HB-*co*-3HHx) biosynthesis by NSDG-GG-based engineered strains of *R. eutropha* from fructose. The amounts of 3HB and 3HHx units in PHA are shown in gray and black bars, respectively. The cells were cultivated in a 100 ml MB medium containing 1% (w/v) fructose for 72 h at 30 °C (n = 3)

*Ralstonia eutropha* strains NSDG-GG-∆B1 harboring either plasmid for P(3HB-*co*-3HHx) biosynthesis accumulated the copolyester from fructose with much higher 3HHx composition of 14.7–21.5 mol% (entries 16 and 22 in Fig. 3 and Additional file 1: Table S2) than the parent *phaB1*^+^ strains (entries 15 and 21), as also seen on glucose. The insertion of *phaB2* into the *phaB1*-deleted chromosome increased the copolyester content up to 73.0–74.1 wt%, of which the 3HHx composition (10.0–10.3 mol%) was intermediate between those by *phaB1*-deleted and *phaB1*^+^ strains (entries 19 and 25). The enhancement of reverse β-oxidation in the *phaB1*-deleted strain increased the 3HHx composition, as the strain harboring *phaJ*_*Ac*_ efficiently produced P(3HB-*co*-13.8 mol% 3HHx) (entry 26). Although these properties were similar to those on glucose, a few different features were observed. One is that the reduction of the cellular PHA content caused by the *phaB1* deletion on fructose was less than that on glucose. Interesting differences were observed in the effects of the low-activity mutants of PhaB1. Unlike on glucose, the insertion of *phaB1*_NV_ or *phaB1*_NVYF_ into the *phaB1*-deleted strain promoted incorporation of the 3HB unit, resulting in an increase of PHA content and a relatively slight decrease of the 3HHx composition on fructose (entries 17, 18, 23, and 24).

### PHA biosynthesis by the engineered *R. eutropha* strains from glycerol

**Fig. 4:**
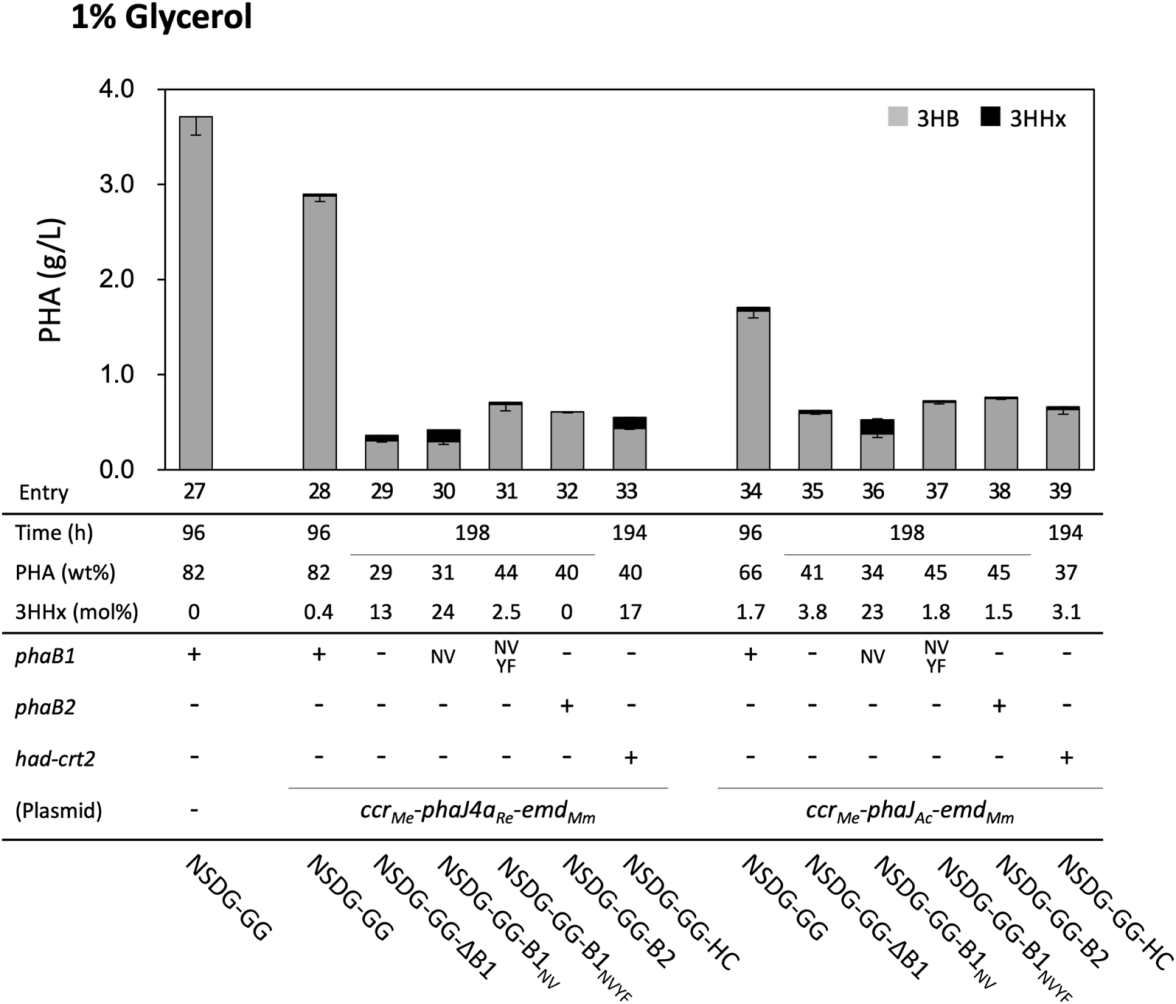
P(3HB-*co*-3HHx) biosynthesis by NSDG-GG-based engineered strains of *R. eutropha* from glycerol. The amounts of 3HB and 3HHx units in PHA are shown in gray and black bars, respectively. The cells were cultivated in a 100 ml MB medium containing 1% (w/v) glycerol for 96 h or 194 h–198 h at 30 °C (n = 3)

The strains NSDG-GG harboring the copolyester biosynthesis plasmid produced P(3HB-*co*-3HHx) from glycerol, although the 3HHx fractions showed faint levels of 0.4–1.7 mol% (entries 28 and 34 in Fig. 4 and Additional file 1: Table S3). In contrast to the cultivation on sugars, the *phaB1* deletion severely impaired PHA biosynthesis from glycerol. When the strain NSDG-GG-∆B1/pBPP-ccr_Me_J4a-emd was cultivated on glycerol, the optical density at 600 nm (OD_600_) increased very slowly and reached to maximum (~ 6) after prolonged cultivation for 196 h (data not shown). This OD_600_ value was about half of those on sugars after 72 h, and the cellular PHA content was only 29.4 wt% (entry 29). Although the 3HHx composition of 13.1 mol% was rather high, this was attributed to a marked decrease in 3HB units. We noticed that this slow increase in OD_600_ on glycerol was not due to slow cell growth, as the residual cell mass after 96 h cultivation on glycerol (0. 81 g/L) was not so markedly less than that on fructose after 72 h (1.10 g/L). Given that the optical density of PHA-producing cells was responsible for both cell concentration and intracellular accumulation of PHA, the slow increase in optical density after 96 h indicated a low rate of PHA synthesis on glycerol caused by the *phaB1* deletion. Further engineered strains showed still poor PHA biosynthesis ability on glycerol despite the insertion of *phaB1*_NV_, *phaB1*_NVYF_, *phaB2*, or *had*-*crt2* (entries 30–33). The impaired ability to biosynthesize PHA could not be restored even by PhaJ_*Ac*_ having high activity to crotonyl-CoA (entries 35-39).

## Discussion

The microbial production of P(3HB-*co*-3HHx) has usually utilized vegetable oils or fatty acids as carbon sources because the provision of (*R*)-3HHx-CoA monomer can be simply achieved by (*R*)-specific hydration of 2-enoyl-CoA intermediate in β-oxidation catalyzed by *R*-hydratase (PhaJ) [12]. The industrial production of P(3HB-*co*-3HHx) from palm oil by recombinant *R. eutropha* has been demonstrated by Kaneka Corp., Japan, since 2011. In addition to vegetable oils, the use of other inexpensive biomass feedstocks, such as sugars and glycerol, is expected to be another promising way of achieving low-cost production and consequent wide applications. From this perspective, we previously engineered *R. eutropha* for the expansion of utilizable carbon sources [28, 32] and for the generation and polymerization of (*R*)-3HHx-CoA from fructose through an artificial pathway [23]. These were here integrated into *R. eutropha*, which led to the construction of strains capable of synthesizing P(3HB-*co*-3HHx) from not only fructose but also glucose and glycerol. However, the yield and 3HHx composition of the copolyesters were insufficient, so further investigation focused on improving the strains to achieve efficient production of the copolyesters with a higher 3HHx fraction.

**Fig. 5:**
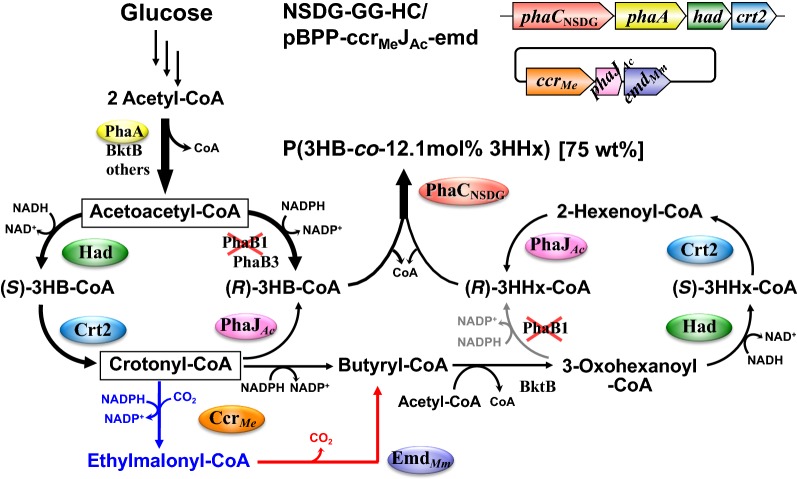
Proposed pathway for P(3HB-*co*-3HHx) biosynthesis from glucose by *R. eutropha* NSDG-GG-HC/pBPP-ccr_Me_J_Ac_-emd. PhaA and BktB, β-ketothiolases; PhaB1 and PhaB3, NADPH-acetoacetyl-CoA reductases; Had, NAD-(*S*)-3HB-CoA dehydrogenase; Crt2, crotonase; PhaC_NSDG_, N149S/D171G mutant of PHA synthase from *A. caviae*; PhaJ_*Ac*_, short-chain-length-(*R*)-enoyl-CoA hydratase from *A. caviae*; Ccr_*Me*_, crotonyl-CoA carboxylase/reductase from *Methylorubrum extorquens*; Emd_*Mm*_, ethylmalonyl-CoA decarboxylase from *Mus musculus*

NADPH-dependent acetoacetyl-CoA reductase (PhaB) is an (*R*)-3HB-CoA-providing enzyme in most P(3HB)-producing microorganisms, and *R. eutropha* possesses three paralogs of PhaB (PhaB1, PhaB2, PhaB). The roles of the multiple PhaBs in *R. eutropha* have been investigated and discussed based on the biosynthesis of P(3HB) homopolymer [14]. PhaB1 from *R. eutropha* has also been frequently applied in PHA biosynthesis by engineered *E. coli* strains. Nevertheless, the effects of modifications in the acetoacetyl-CoA reduction step on the biosynthesis of PHA copolymers have not been well considered. In the case of P(3HB-*co*-3HHx) biosynthesis from fructose by the previously engineered *R. eutropha* [23], deletion of *phaB1* was an important modification for the incorporation of 3HHx unit into the polyester fraction, although this accompanied the reduction of PHA production. It was supposed that (*R*)-3HB-CoA provision in the *phaB1*-lacking strain, supported by the minor paralog PhaB3 (associated with only 2%–5% of total NADPH-dependent acetoacetyl-CoA reductase activity in cell extracts of *R. eutropha* [14, 33]), was significantly weakened when compared with the parent *phaB1*^+^-strains. This tradeoff between production and composition of the copolyesters depending on the presence or absence of PhaB1 coincided with the formation of butyryl-CoA mainly via (*S*)-3HB-CoA, and suggested the importance of flux distribution at the acetoacetyl-CoA node for the copolyester biosynthesis through the artificial pathway. We assumed that moderate weakening of the (*R*)-specific reduction of acetoacetyl-CoA would establish metabolic flux distribution from acetoacetyl-CoA to (*R*)- and (*S*)-3HB-CoAs suitable for P(3HB-*co*-3HHx) synthesis. The *R. eutropha* strains were thus modified by introducing low-affinity mutants (PhaB1_NV_ and PhaB1_NVYF_) and a low-activity paralog (PhaB2) of PhaB1 for moderate weakening of the (*R*)-specific reduction. On glucose, the use of PhaB2 instead of PhaB1 resulted in production of the copolyester with cellular content and 3HHx composition in-between those by the *phaB1*^+^ and ∆*phaB1* strains, whereas the two kinds of low-affinity mutant of PhaB1 did not significantly affect PHA biosynthesis. These results demonstrated that the flux distribution from acetoacetyl-CoA toward C_4_- and C_6_-monomers can be regulated by specific activity levels of the reductase when the enzyme retains high affinity to acetoacetyl-CoA. As previous metabolomic analysis of *R. eutropha* showed a very low intracellular concentration of acetoacetyl-CoA [40], high substrate affinity of the reductase was required to change the metabolic fluxes. Another idea to overcome the tradeoff was the enhancement of reverse β-oxidation forming 2-enoyl-CoAs from 3-oxoacyl-CoAs via (*S*)-3HA-CoAs. The introduction of the second copies of *had* and *crt2* into the *pha* operon in the ∆*phaB1* strain increased the 3HHx composition of the polyester fraction without a serious negative impact on PHA production. The combination of the enhanced reverse β-oxidation and weakened (*R*)-specific reduction possibly increased butyryl-CoA formation from acetoacetyl-CoA and the following elongation to 3-oxohexanoyl-CoA, as well as the next reverse cycle to (*R*)-3HHx-CoA (Fig. 5). As seen on vegetable oils [18], the copolyester biosynthesis was also affected by the substrate specificity of *R*-hydratase. The use of short-chain-length-specific PhaJ_*Ac*_ tended to increase the C_4_ units and decreas the C_6_ units when compared with medium-chain-length-specific PhaJ4a. This was because crotonyl-CoA was the second node for redistribution of the flux to the C_4_- and C_6_-monomers. When PhaJ_*Ac*_ was functional, crotonyl-CoA was partially intercepted to form (*R*)-3HB-CoA and thus the decrease in the C_4_ units caused by the lack of PhaB1 was compensated to some extent (Fig. 5). Wang et al. reported on the biosynthesis of P(3HB-*co*-3HHx) from glucose by recombinant *E. coli* strains harboring *trans*-2-enoyl-CoA reductase from *Treponema denticola* in BktB-dependent condensation pathway (14.2 wt%, 4.0 mol% 3HHx) or reverse β-oxidation pathway using FadBA from *E. coli* (12.4 wt%, 10.2 mol% 3HHx) [41]. In this study, the practically useful P(3HB-*co*-12.1 mol% 3HHx) could be produced with cellular content of 75.3 wt% from glucose by the engineered *R. eutropha* strain NSDG-GG-HC/pBPP-ccr_Me_J_Ac_-emd.

The modifications of the acetoacetyl-CoA reduction step had similar effects on P(3HB-*co*-3HHx) biosynthesis from fructose to those from glucose (Fig. 3 and Additional file 1: Table S2), although differences were observed when the PhaB1 mutants were introduced. On fructose, the recombinant strains having PhaB1_NV_ or PhaB1_NVYF_ accumulated more PHA with a larger 3HB fraction than the ∆*phaB1* strain, which was not seen on glucose. This result strongly suggested actual functions of the PhaB1 mutants in (*R*)-3HB-CoA formation on fructose, despite the low affinity to acetoacetyl-CoA. This might be due to altered metabolic regulation depending on the sugar uptake machinery. In the *R. eutropha* H16-derived strains, fructose is incorporated and 6-phosphorylated by ATP-binding cassette transporter (FrcACB) [42] and fructokinase, respectively, while the uptake and 6-phosphorylation of glucose is mediated by mutated GlcNAc-specific PTS (NagFE) [28]. It is well known that glucose-specific PTS is associated with catabolite repression in *E. coli*. Likewise, the glucose-transportation by the mutant of NagFE in the *R. eutropha* strains may affect gene expression and consequent changes in metabolic regulation, such as a further decrease in intracellular pool of acetoacetyl-CoA, toward which catalytic functions of the low-affinity PhaB1 mutants were limited.

The engineered strain NSDG-GG could grow and accumulate P(3HB) well on glycerol, whereas the deletion of *phaB1* severely impaired PHA biosynthesis from glycerol, unlike from sugars. It has been demonstrated that PhaB1 and PhaB3 contributed to P(3HB) biosynthesis on fructose, while only PhaB1 had a role on palm oil [14]. This was probably due to weak expression of *phaB3* on palm oil as shown by microarray analysis [35]. The present results strongly suggested that *phaB3* expression was tightly repressed on not only vegetable oils but also another non-sugar substrate, glycerol. We further observed that introduction of the genes of PhaB1 mutants, PhaB2, or Had-Crt2 into the ∆*phaB1* strain only faintly restored the PHA biosynthesis. The highly efficient and highly expressed PhaB1 was essential to convert acetoacetyl-CoA to (*R*)-3HB-CoA under the conditions on glycerol. A further engineering strategy to enhance the C_6_ unit-formation pathway even with the functions of PhaB1 should be investigated to achieve the production of P(3HB-*co*-3HHx) from glycerol. Such strategy is also expected to be useful for more efficient production of PHA copolyesters and other compounds by *phaB1*^+^-strains from various structurally unrelated carbon sources.

## Conclusions

We herein engineered *Ralstonia eutropha* for the biosynthesis of P(3HB-*co*-3HHx) from structurally unrelated glucose and glycerol by conferring glucose assimilation ability, enhancing glycerol assimilation ability, and installing an artificial pathway for biosynthesis of the copolyester. Further modifications at the acetoacetyl-CoA reduction step demonstrated the importance of flux distribution at the acetoacetyl-CoA node in *R. eutropha* for the biosynthesis of the PHA copolyesters with regulated composition from the structurally unrelated compounds. The moderate weakening of the (*R*)-specific reduction of acetoacetyl-CoA or enhancement of reverse β-oxidation allowed efficient biosynthesis of the copolyesters from glucose with high 3HHx composition, possibly due to enhanced formation of butyryl-CoA, a precursor of the C_6_-intermediates, from acetoacetyl-CoA via (*S*)-3HB-CoA. This study can provide important information for the engineering of *R. eutropha* for the production of PHAs as well as other acetyl-CoA-derived compounds.

## Methods

### Bacterial strains and plasmids

The strains and plasmids used in this study are listed in Table 2. *Escherichia coli* strains DH5α and S17-1 [43], used as a host strain for general genetic engineering and transconjugation, respectively. *R. eutropha* strains were routinely cultivated at 30 °C in a nutrient-rich (NR) medium containing 1% (w/w) meat extract, 1% (w/v) polypeptone, 0.2% (w/w) yeast extract dissolved in tap water. Kanamycin (100 µg/mL for *E. coli* and 250 µg/mL for *R. eutropha* strains) or ampicillin (100 µg/mL for *E. coli*) was added into the medium when necessary.

**Table 2:** Strains and plasmids used in this study

Strain or plasmid	Relevant marker	Source or references
*Escherichia coli*		
DH5*α*	F^−^, *ϕ*80*lacZ*∆M15, ∆(*lacZYA*-*argF*) U169, *deoR*, *recA*1, *endA*1, *hsdR*17(r_K_^−^ m_K_^+^), *phoA*, *supE*44, λ^−^, *thi*-1, *gyrA*96, *relA*1	Lab stock
BL21(DE3)	*E. coli* B, F^−^, *dcm*, *ompT*, hsdS(r_B_^−^ m_B_^−^), gal, λ(DE3)	Novagen
S17-1	*thi pro hsdR recA* chromosomal RP4; Tra^+^; Tmp^r^ Str/Spc^r^	[43]
*Ralstonia eutropha*		
H16	Wild type	DSM 428
NSDG	H16 derivative; Δ*phaC*::*phaC*_NSDG_	[33]
NSDG-GG	NSDG derivative; Δ*nagR*, *nagE*(G793C), *P*_A2858_-*glpFK*_*Ec*_-*h16_A2858*	This study
NSDG-GGΔB1	NSDG-GG derivative; Δ*phaB1*	This study
NSDG-GG-B_NV_	NSDG-GG∆B1 derivative; *phaB1*_NV_	This study
NSDG-GG-B_NVYF_	NSDG-GG∆B1 derivative; *phaB1*_NVYF_	This study
NSDG-GG-B2	NSDG-GG∆B1 derivative; ∆phaB1::*phaB*2	This study
NSDG-GG-HC	NSDG-GG∆B1 derivative; ∆phaB1::*had*-*crt2*	This study
Plasmids		
pEE32	pUC18 derivative; *phaPCJ*_*Ac*_	[10]
pBPP	pBBR ori (broad host range), *mob*, Kan^r^, *P*_*phaP1*_	[44]
pBPP-ccr_Me_J4a-emd	pBPP derivative; *ccr*_*Me*_, *phaJ4a*, *emd*_*Mm*_	[23]
pBPP-ccr_Me_J_Ac_-emd	pBPP derivative; *ccr*_*Me*_, *phaJ*_*Ac*_, *emd*_*Mm*_	This study
pColdII	ColE1 ori, Amp^r^, *P*_*cspA*_	Takara Bio
pColdII-phaB1	pColdII derivative, *phaB1*	This study
pColdII-phaB2	pColdII derivative, *phaB2*	This study
pET15b-phaB3	pET15b derivative, *phaB3*	This study
pColdII-phaB1_NV_	pColdII derivative, *phaB1*_NV_	This study
pColdII-phaB1_NVYF_	pColdII derivative, *phaB1*_NVYF_	This study
pK18mobsacB-AR	pK18mobsacB derivative; *phaB1 del*	[33]
pK18mobsacB-AB_NVYF_R	pK18mobsacB-AR derivative; *phaB1*_NVYF_ *ins*	This study
pK18mobsacB-AB_NV_R	pK18mobsacB-AR derivative; *phaB1*_NV_ *ins*	This study
pK18mobsacB-AB2R	pK18mobsacB-AR derivative; *phaB2 ins*	This study
pK18mobsacB-AHCR	pK18mobsacB-AR derivative; *had*-*crt2 ins*	This study

The postfix *del* and *ins* indicate constructs for targeted gene deletion and insertion, respectively

*Ac*, *Aeromonas caviae*; *Me*, *Methylorubrum extorquen*s; *Mm*, *Mus musculus*. *phaC*_NSDG_, a gene encoding N149S/D171G mutant of PHA synthase from *A. caviae. phaB1*_NV_ and *phaB1*_NVYF_, genes encoding N142V and N142V/Y185F mutants of NADPH-acetoacetyl-CoA reductase PhaB1 from *R. eutropha*, respectively

### Plasmid construction

DNA manipulations were carried out according to standard procedures, and PCR reactions were performed with KOD-Plus ver.2 DNA polymerase (Toyobo, Osaka). The sequences of oligonucleotide primers used in this study are shown in Additional file 1: Table S4.

pColdII-phaB1, pColdII-phaB2, and pET15b-phaB3 vectors for overproduction of *N*-His_6_-tagged PhaB1, PhaB2, and PhaB3 in *E. coli*, respectively, were constructed as described in Supporting Information. Site-directed mutagenesis of *phaB1* was carried out by QuickChange protocol. A DNA fragment consisting of a tandem of *had* and *crt2* was prepared by fusion PCR. Several plasmids were constructed by blunt-end ligation of a DNA fragment with a linear fragment of the backbone plasmid prepared by inverse PCR. pBPP-ccr_Me_J_Ac_-emd was constructed by replacement of *phaJ4a* in pBPP-ccr_Me_J4a-emd [23, 44] by *phaJ*_*Ac*_ amplified with pEE32 [10] as a template. Plasmids for homologous recombination for insertion of the mutagenized genes of *phaB1*, *phaB2*, or *had*-*crt2* into chromosome 1 of *R. eutropha* at the *phaB*1 locus were constructed from pK18mobsacB-AR previously made for deletion of *phaB1* [33]. The target genes were individually inserted into pK18mobsacB-AR as located downstream of *phaA* with the same orientation.

### Preparation of *N*-His_6_-tagged recombinant proteins and enzyme assay

Overexpression of *phaB* genes in *E. coli* BL21(DE3) harboring the expression plasmids with IPTG induction, and purification of the *N*-His_6_-tagged recombinant proteins using Ni-affinity chromatography were carried out as described in Supporting Information. NADPH-dependent acetoacetyl-CoA reductase activity was assayed in the mixture composed of 200 μM NADPH, 1 to 20 μM acetoacetyl-CoA (Sigma-Aldrich, St. Louis, MO, USA), and enzyme solution with appropriate dilution in 200 μL of 100 mM Tris–HCl buffer (pH 8.0). The consumption of NADPH accompanied by decrease in absorbance at 340 nm was monitored at 30 °C (ε_340_ = 6.22 × 10^3^ M^−1^ cm^−1^).

### Construction of recombinant strains of *R. eutropha*

Transformation of *R. eutropha* was carried out by transconjugation using *E. coli* S17-1 harboring a pK18mobsacB-based plasmid as a donor, and transformants generated by pop in-pop out recombination were isolated as described previously [20, 33]. *R. eutropha* strain NSDG-GG was constructed by sequential chromosomal modifications for glucose assimilation [28] and enhanced glycerol assimilation [32] into strain NSDG [33]. The strain NSDG-GG-∆B1 was constructed by deletion of *phaB1* in NSDG-GG using pK18mobsacB-AR, and the other strains NSDG-GG-B_NV_, NSDG-GG-B_NVYF_, NSDG-GG-B2, and NSDG-GG-HC were obtained by insertion of *phaB1*_NV_, *phaB1*_NVYF_, *phaB2*, and *had*-*crt2*, respectively, into NSDG-GG-∆B1 at downstream of *phaA* using the corresponding plasmids.

### PHA production

*Ralstonia eutropha* strains were cultivated at 30 °C in 100 mL of a nitrogen-limited mineral salts (MB) medium composed of 0.9 g of Na_2_HPO_4_•12H_2_O, 0.15 g of KH_2_PO_4_, 0.05 g of NH_4_Cl, 0.02 g of MgSO_4_•7 H_2_O, and 0.1 ml of trace-element solution [45] in 100 ml of deionized water. A filter-sterilized solution of glucose, fructose, or glycerol was added to the medium at a final concentration of 1.0% (*w/v*) as a sole carbon source. Kanamycin was added at the final concentration of 300 µg/mL. After the cultivation for 72 h with reciprocal shaking (115 strokes/min), the cells were harvested, washed once with cold deionized water, and then lyophilized. The cellular PHA content and composition were determined by gas chromatography (GC) after direct methanolysis of the dried cells in the presence of 15% sulfuric acid as described previously [45].

## Supplementary information

**Additional file 1.** Additional figures and tables.

## Data Availability

All data generated or analyzed during this study are included in this published article.
