# Supplementary material for: Modification of acetoacetyl-CoA reduction step in Ralstonia eutropha for biosynthesis of poly(3-hydroxybutyrate-co-3-hydroxyhexanoate) from structurally unrelated compounds
Source: Microb Cell Fact. 2019 Aug 29;18:147. doi: 10.1186/s12934-019-1197-7 (PMC6716841; doi:10.1186/s12934-019-1197-7)
Supplement: Supplementary file 1 — Additional file 1. Additional figures and tables. [file 12934_2019_1197_MOESM1_ESM.pdf]

Additional file 1:

Modification of acetoacetyl-CoA reduction step in *Ralstonia eutropha* for biosynthesis of poly(3-hydroxybutyrate-*co*-3-hydroxyhexanoate) from structurally unrelated compounds

Mengxiao Zhang, Shunsuke Kurita, Izumi Orita, Satoshi Nakamura, Toshiaki Fukui*

School of Life Science and Technology, Tokyo Institute of Technology

*Corresponding author
Toshiaki Fukui
School of Life Science and Technology
Tokyo Institute of Technology
B-37 4259 Nagatsuta, Midori-ku, Yokohama 226-8501, Japan
Tel/Fax: +81-45-924-5766
e-mail: tfukui@bio.titech.ac.jp

Supporting Information

Fig. S1
Fig. S2
Table S1
Table S2
Table S3
Table S4

Construction of expression plasmids for *N*-His$_6$-tagged PhaBs

The coding regions of *phaB1*, *phaB2*, and *phaB3* were amplified by PCR using primer sets shown in Supplementary Table S4 and *R. eutropha* gDNA, and the amplified fragments were individually cloned into pUC118. The *phaB1* fragment was excised by digestion with NdeI and BamHI, and then inserted into pCold II (Takara Bio) at the corresponding sites to obtain pColdII-phaB1. pET15b-phaB3 was constructed by insertion of an NdeI-BamHI-restricted fragment of *phaB3* into pET15b (Novagen), because the gene expression using a pCold II-based vector was not observed by unknown reason. The *phaB2* fragment was excised by KpnI and inserted into pCold II at the corresponding site. The extra region between the His$_6$-tag sequence and *phaB2* was removed by inverse PCR and successive self-ligation to obtain pColdII-phaB2.

Preparation of *N*-His$_6$-tagged recombinant proteins

E. coli BL21(DE3) transformed with pColdII-phaB1 or pColdII-phaB2 was cultivated in LB medium at 37˚C on a reciprocal shaker (115 strokes/min). When the cell growth reached to OD_{600} of 0.4, the culture broth was cooled at 15˚C for 30 min and IPTG was added at the final concentration of 0.5 mM for induction of gene expression. The cultivation was continued for further 24 h at 15˚C. In the case of expression of *phaB3*, *E. coli* BL21(DE3) harboring pET15b-phaB3 was cultivated in LB medium at 37˚C on a reciprocal shaker and the gene expression was induced by addition of 0.5 mM IPTG when OD_{600} reached to 0.5, and the cells were cultivated for further 16 h at 37˚C.

The cells were harvested, washed and resuspended within 20 mM sodium phosphate buffer (pH7.4) containing 0.5 M NaCl and 30 mM imidazole, and then disrupted by sonication. The soluble fraction prepared by centrifugation and filtration (pore size 0.20 μm) was subjected to Ni-affinity chromatography using HisTrap FF crude 1 mL (GE Healthcare Life Sciences). The *N*-terminal His$_6$-tagged recombinant proteins were eluted by linear gradient of imidazole from 30 mM to 500 mM in sodium phosphate buffer (pH7.4) containing 0.5 M NaCl. The protein fraction was desalted using HiTrap Desalting (GE Healthcare Life Sciences) with 50 mM Tris-HCl (pH7.5), and then used for enzyme assay.

Site-directed mutagenesis of PhaB1

NADPH-acetoacetyl-CoA reductase (PhaB) is belonging to a short-chain dehydrogenase/ reductase family along with NADPH-3-oxoacyl-ACP reductase (FabG). It is known that the former shows strict specificity to acetoacetyl-CoA, while the latter can accept 3-oxoacyl-ACPs with medium-chain-length as substrates. We had attempted protein engineering of PhaB1 based

on comparison of the crystal structures of PhaB1 and FabG, aiming to obtain the enzyme capable of catalyzing (*R*)-specific reduction of the C_6 substrate, 3-oxohexanoyl-CoA.

The sequence alignment of three PhaB paralogs from *R. eutropha* (PhaB1, PhaB2, and PhaB3) [1] and FabGs from *E. coli* and *Pseudomonas* sp. 61-3 [2] identified residues that were conserved in the three PhaBs but not in FabGs, and comparison of the three-dimensional structures of FabG from *E. coli* complexed with $NADP^+$ (1Q7B) [3] and PhaB1 complexed with $NADP^+$ (not containing the substrate, obtained by personal communication from Hokkaido Univ., before deposition of that complexed with $NADP^+$ and acetoacetyl-CoA (3VZS) [4]). This suggested that a cavity near from the probable substrate binding pocket was filled by the side chains of asparagine 142 and tyrosine 185 in PhaB1 (Supplementary Fig. S2) possibly forming a hydrogen bond. We therefore replaced these residues by valine and phenylalanine correspondingly conserved in FabGs, respectively.

The site directed mutagenesis was carried out by QuickChange protocol using primer sets N142V_Fw/N142V_Re and Y185F_Fw/Y185F_Re for N142V and Y185F mutations, respectively. The NdeI-BamHI restricted fragments of the mutagenized genes were inserted into pCold II at the corresponding sites. The *N*-terminal His_6-tagged PhaB1s with N142V, Y185F, or N142V/Y185F mutations (designated as $PhaB1_{NV}$, $PhaB1_{YF}$, and $PhaB1_{NVYF}$, respectively) were produced by recombinant strains of *E. coli* BL21(DE3) harboring each the expression plasmid, and then purified by the same procedure for PhaB1 as described in the main text. NADPH-dependent reduction activities towards acetoacetyl-CoA and 3-oxohexanoyl-CoA were determined as described previously [5].

Unfortunately, expansion of the substrate specificity was not achieved by the site-directed mutagenesis, since the ratios of activity to the C_6 and C_4 substrates of the three mutants were 0.020-0.023 (C_6/C_4) that was only slightly higher than 0.013 of the parent wild-type enzyme. Further characterization of the mutants clarified that catalytic efficiencies for NADPH-dependent reduction of acetoacetyl-CoA were markedly reduced by the mutations (Table 2 in the main text). When compared to wild-type PhaB1, the Km values of the single mutants $PhaB1_{NV}$ and $PhaB1_{YF}$ were remarkably larger (58μM–86 μM), while the Vmax values were similar for $PhaB1_{NV}$ and slightly lower for $PhaB1_{YF}$. The double mutant $PhaB1_{NVYF}$ exhibited the lowest affinity and reaction rate toward acetoacetyl-CoA among the three mutants.

References

1. Pohlmann A, Fricke WF, Reinecke F, Kusian B, Liesegang H, Cramm R, Eitinger T, Ewering C, Potter M, Schwartz E, Strittmatter A, Voss I, Gottschalk G, Steinbuchel A, Friedrich B, Bowien B. Genome sequence of the bioplastic-producing "Knallgas" bacterium *Ralstonia eutropha* H16. Nat Biotechnol. 2006;24:1257-62.

2. Nomura CT, Taguchi K, Gan Z, Kuwabara K, Tanaka T, Takase K, Doi Y. Expression of 3-ketoacyl-acyl carrier protein reductase (*fabG*) genes enhances production of polyhydroxyalkanoate copolymer from glucose in recombinant *Escherichia coli* JM109. Appl Environ Microbiol. 2005;71:4297-306.

3. Price AC, Zhang YM, Rock CO, White SW. Cofactor-induced conformational rearrangements establish a catalytically competent active site and a proton relay conduit in FabG. Structure. 2004;12:417-28.

4. Matsumoto K, Tanaka Y, Watanabe T, Motohashi R, Ikeda K, Tobitani K, Yao M, Tanaka I, Taguchi S. Directed evolution and structural analysis of NADPH-dependent acetoacetyl coenzyme A (acetoacetyl-CoA) reductase from *Ralstonia eutropha* reveals two mutations responsible for enhanced kinetics. Appl Environ Microbiol. 2013;79:6134-9.

5. Segawa M, Wen C, Orita I, Nakamura S, Fukui T. Two NADH-dependent (*S*)-3-hydroxyacyl-CoA dehydrogenases from polyhydroxyalkanoate-producing *Ralstonia eutropha*. J Biosci Bioeng. 2019;127:294-300.

(A) Glucose transport and phosphorylation

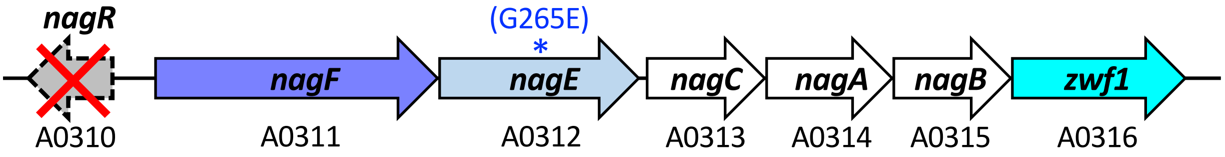

(B) Glycerol transport and phosphorylation

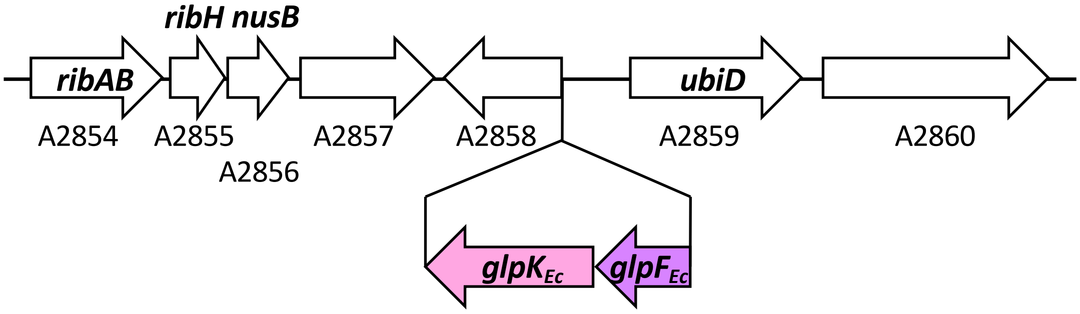

Fig. S1. Modifications of chromosome 1 of *R. eutropha* H16 for assimilation of glucose (A) [Orita I, et al. J Biosci Bioeng. 2011;113:63-9], and enhanced assimilation of glycerol (B) [Fukui T, et al. Appl Microbiol Biotechnol. 2014;98:7559-68.].

(A) PhaB1 from *R. eutropha* (B) FabG from *E. coli*

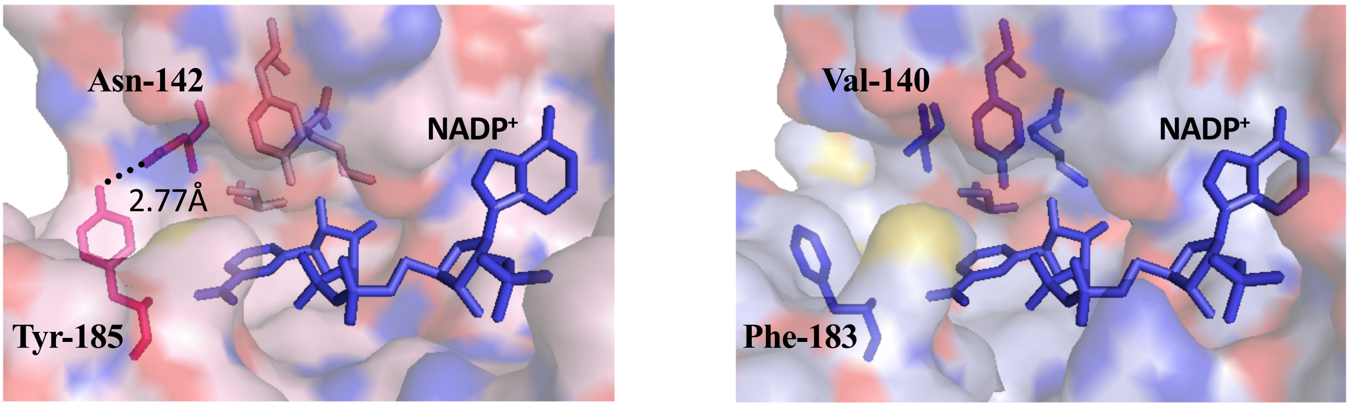

Fig. S2. Structure of $NADP^+$-binding sites in PhaB1 from *R. eutropha* (A) and FabG from *E. coli* (B). Side chains of amino acid residues in interest and $NADP^+$ are shown as stick models.

Table S1. P(3HB-*co*-3HHx) biosynthesis by NSDG-GG-based engineered strains of *R. eutropha* from glucose

Entry	Strain	Plasmid	Cultivation time (h)	Dry cell mass (g/L)	PHA content (wt%)	PHA (g/L)	3HHx (mol%)	Monomer amount in PHA [mmol/l-culture]	
								3HB	3HHx
1	NSDG-GG	none	72	4.56 ±0.14	83.1 ±2.6	3.78 ±0.01	0	44.0 ±0.1	0
2	NSDG-GG	pBPP-ccr$_{Me}$J4a-emd	72	4.09 ±0.02	80.1 ±0.4	3.28 ±0.04	2.3 ±0.2	36.9 ±0.5	0.9 ±0.1
3	NSDG-GG-ΔB1			2.74 ±0.15	62.5 ±3.3	1.71 ±0.16	22.0 ±0.6	14.5 ±1.2	4.1 ±0.5
4	NSDG-GG-B$_{NV}$			2.66 ±0.10	58.7 ±0.7	1.56 ±0.07	15.3 ±0.1	14.7 ±0.7	2.6 ±0.1
5	NSDG-GG-B$_{NVYF}$			2.93 ±0.03	63.6 ±0.8	1.86 ±0.01	12.0 ±0.9	18.3 ±0.2	2.5 ±0.2
6	NSDG-GG-B2			3.96 ±0.01	72.3 ±2.5	2.86 ±0.09	5.9 ±0.1	30.7 ±1.0	1.9 ±0.1
7	NSDG-GG-HC			2.99 ±0.24	68.7 ±0.2	2.05 ±0.16	26.0 ±0.4	16.3 ±1.1	5.7 ±0.5
8	NSDG-GG	pBPP-ccr$_{Me}$J$_{Ac}$-emd	72	3.42 ±0.05	76.1 ±0.4	2.60 ±0.04	4.1 ±0.2	28.6 ±0.6	1.2 ±0.1
9	NSDG-GG-ΔB1			2.76 ±0.04	62.8 ±0.8	1.74 ±0.01	8.3 ±0.2	18.0 ±1.6	1.6 ±0.2
10	NSDG-GG-B$_{NV}$			3.06 ±0.02	69.9 ±1.1	2.14 ±0.03	8.7 ±0.3	22.1 ±0.3	2.1 ±0.1
11	NSDG-GG-B$_{NVYF}$			3.00 ±0.13	66.7 ±2.0	2.00 ±0.15	11.2 ±0.4	19.9 ±1.5	2.5 ±0.2
12	NSDG-GG-B2			3.65 ±0.03	71.4 ±0.3	2.61 ±0.01	7.0 ±0.2	27.6 ±0.2	2.1 ±0.0
13	NSDG-GG-HC			3.71±0.13	75.3 ±0.4	2.79 ±0.08	12.1 ±0.3	27.5 ±0.9	3.8 ±0.0

The cells were cultivated in a 100 ml MB medium containing 1% (w/v) glucose for 72 h at 30˚C.
Standard deviation was shown with each value (*n*=3).

Table S2. P(3HB-*co*-3HHx) biosynthesis by NSDG-GG-based engineered strains of *R. eutropha* from fructose.

Entry	Strain	Plasmid	Cultivation time (h)	Dry cell mass (g/L)	PHA content (wt%)	PHA (g/L)	3HHx (mol%)	Monomer amount in PHA [mmol/l-culture]	
								3HB	3HHx
14	NSDG-GG	none	72	3.76 ±0.03	81.2 ±0.5	3.05 ±0.04	0	35.5 ±0.5	0
15	NSDG-GG	pBPP-ccr$_{Me}$J4a-emd	72	3.05 ±0.03	75.2 ±0.5	2.29 ±0.04	0.9 ±0.1	26.4 ±0.4	0.2 ±0.0
16	NSDG-GG-ΔB1			2.99 ±0.11	63.3 ±1.0	1.90 ±0.09	21.5 ±0.9	16.2 ±0.9	4.4 ±0.2
17	NSDG-GG-B$_{NV}$			3.38 ±0.04	71.5 ±1.7	2.42 ±0.08	14.8 ±0.4	22.8 ±0.8	4.0 ±0.2
18	NSDG-GG-B$_{NVYF}$			3.70 ±0.08	73.3 ±1.9	2.71 ±0.13	13.3 ±0.1	26.2 ±1.2	4.0 ±0.2
19	NSDG-GG-B2			3.90±0.02	73.0 ±1.8	2.85 ±0.06	10.3 ±0.3	28.8 ±0.7	3.3 ±0.1
20	NSDG-GG-HC			2.76 ±0.12	74.5 ±1.0	2.06 ±0.11	24.2 ±0.8	16.8 ±1.1	5.4 ±0.1
21	NSDG-GG	pBPP-ccr$_{Me}$J$_{Ac}$-emd	72	2.68 ±0.03	69.6 ±2.2	1.87 ±0.08	2.1 ±0.1	21.1 ±1.0	0.5 ±0.0
22	NSDG-GG-ΔB1			2.93 ±0.03	64.1 ±0.3	1.87 ±0.01	14.7 ±0.3	17.8 ±0.1	3.1 ±0.1
23	NSDG-GG-B$_{NV}$			3.52 ±0.07	75.0 ±0.8	2.64 ±0.06	9.4 ±0.3	26.9 ±0.7	2.8 ±0.1
24	NSDG-GG-B$_{NVYF}$			3.67 ±0.10	72.5 ±3.9	2.66 ±0.11	10.8 ±0.5	26.6 ±1.0	3.2 ±0.3
25	NSDG-GG-B2			3.78±0.05	74.1±1.9	2.80 ±0.07	10.0 ±0.3	28.4 ±0.6	3.2 ±0.1
26	NSDG-GG-HC			3.60±0.31	78.1 ±0.6	2.81 ±0.23	13.8 ±0.3	27.0 ±2.3	4.3 ±0.3

The cells were cultivated in a 100 ml MB medium containing 1% (w/v) fructose for 72 h at 30˚C.
Standard deviation was shown with each value (*n*=3).

Table S3. P(3HB-*co*-3HHx) biosynthesis by NSDG-GG-based engineered strains of *R. eutropha* from glycerol.

Entry	Strain	Plasmid	Cultivation time (h)	Dry cell mass (g/L)	PHA content (wt%)	PHA (g/L)	3HHx (mol%)	Monomer amount in PHA [mmol/l-culture]	
								3HB	3HHx
27	NSDG-GG	none	96	4.53 ±0.26	81.8 ±1.8	3.71 ±0.29	0	43.1 ±3.4	0
28	NSDG-GG	pBPP-ccr$_{Me}$J4a-emd	96	3.53 ±0.04	82.0 ±0.8	2.89 ±0.06	0.4 ±0.1	33.5 ±0.7	0.1 ±0.0
29	NSDG-GG-ΔB1		198	1.23 ±0.02	29.4 ±0.4	0.36 ±0.01	13.1 ±0.3	3.5 ±0.1	0.5 ±0.0
30	NSDG-GG-B$_{NV}$			1.35 ±0.01	30.9 ±2.1	0.42 ±0.03	24.0 ±1.2	3.4 ±0.3	1.1 ±0.0
31	NSDG-GG-B$_{NVYF}$			1.62 ±0.15	43.9 ±1.1	0.71 ±0.08	2.5 ±0.1	8.0 ±0.9	0.2 ±0.0
32	NSDG-GG-B2			1.51±0.02	40.2 ±0.6	0.61±0.01	0.0	7.1 ±0.1	0.0
33	NSDG-GG-HC		194	1.37 ±0.03	40.2 ±0.5	0.55 ±0.02	16.6 ±0.1	5.1 ±0.2	1.0 ±0.0
34	NSDG-GG	pBPP-ccr$_{Me}$J$_{Ac}$-emd	96	2.58 ±0.05	66.2 ±1.6	1.71 ±0.07	1.7 ±0.1	19.4 ±0.8	0.3 ±0.0
35	NSDG-GG-ΔB1		198	1.53 ±0.01	40.8 ±0.6	0.62 ±0.01	3.8 ±0.0	6.9 ±0.1	0.3 ±0.0
36	NSDG-GG-B$_{NV}$			1.57 ±0.11	33.5 ±1.2	0.53 ±0.06	22.8 ±0.4	4.4 ±0.5	1.3 ±0.1
37	NSDG-GG-B$_{NVYF}$			1.60 ±0.03	45.2 ±0.2	0.72 ±0.02	1.8 ±0.1	8.2 ±0.2	0.2 ±0.0
38	NSDG-GG-B2			1.71±0.01	44.7±0.9	0.76±0.02	1.5 ±0.0	8.7 ±0.2	0.1 ±0.0
39	NSDG-GG-HC		194	1.79±0.12	36.8 ±0.4	0.66 ±0.05	3.1 ±0.1	7.4 ±0.6	0.2 ±0.0

The cells were cultivated in a 100 ml MB medium containing 1% (w/v) glycerol for 96 h or 194-198 h at 30˚C.
Standard deviation was shown with each value (*n*=3).

Table S4. The sequences of primers used in this study.

Primer	Sequence (5'-3')	Note[a]
Construction of pBPP-ccr$_{Me}$J$_{Ac}$-emd		
phaJAc-Fw	ATGAGCGCACAATCCCTGGAAGTAG	Amplification of *phaJ$_{Ac}$*
phaJAc-Rv	TTAAGGCAGCTTGACCACGGCTTC	
phaJ4a-Inv-1	GTCGATAGTCTCCTCTTGACGATAAAGC	Inverse PCR of pBPP-ccr$_{Me}$J4a-emd
phaJ4a-Inv-2	GGATCCGTTTTTTTGGGCTAGCAGGAGGA	
Construction of *phaB*-expression plasmids		
PhaB1_ESN-N	GGAATTCAAGGAGTGCATATGACTCAGCGCATTGCGTATG	Amplification of *phaB1*
PhaB1_BE-C	GGAATTCGGATCCTCAGCCCATGTGCAGGCCGCCGTTGAG	
PhaB2_KS-N	CGGGTACCAAGGAGCTGGACATGGCCGGACAACGCATTGCC	Amplification of *phaB2*
PhaB2_KE-C	CGGGTACCGGATCCTCATTGCAGGTGTTGCCCGCCGTTGA	
PhaB3_ESN-N	GGAATTCAAGGAGTGCATATGATGAAGAAAATTGCACTGG	Amplification of *phaB3*
PhaB3_BE-C	GGAATTCGGATCCTTACTGCATGTGCTGCCCGCCATTGAT	
phaB2_inv_his	GTGGTGGTGATGATGATGCACTTTGTGA	Inverse PCR of *phaB2*-inserted plasmid
phaB2_inv_pha	ATGGCCGGACAACGCATTGCCCTGGTAA	
Site-directed mutagenesis of PhaB1		
N142V_Fw	CATCTCGTCGGTGgtCGGGCAGAAGGGCC	N142V mutation
N142V_Re	GGCCCTTCTGCCCGacCACCGACGAGATG	
Y185F_Fw	CGGTCTCTCCGGGCTtTATCGCCACCGAC	Y185F mutation
Y185F_Re	GTCGGTGGCGATAaAGCCCGGAGAGACCG	
Construction of plasmids for homologous recombination in *R. eutropha*		
pk18inv_Fw	CCGGCCTGGTTCAACCAGTCGGCA	Inverse PCR of pK18mobsacB-AR
del-phaB1-Inv-3	GTCCACTCCTTGATTGGCTTCGTTA	
phaB1_Fw_ATG	ATGACTCAGCGCATTGCGTATGTG	Amplification of mutagenized *phaB1*
phaB1_Rv	CAGGTCAGCCCATATGCAGGCCGC	
phaB2-Fw	ATGGCCGGACAACGCATTGCCCTGGTAA	Amplification of *phaB2*
phaB2-Rv	TCATTGCAGGTGTTGCCCGCCGTTGAT	
A0602-F	ATGCAAATCCAAGGCAACGTATTCA	Amplification and fusion of *had* and *crt2*
A0602-R-Fus	ATGTATTTGCCTTTACTTGGGCTGCATCCGGA	
A3307-R	GCCTTAGCGATGCTGGAAATT	
A3307-F-Fus	CCAAGTAAAGGCAAATACATAGGAGAAGACA	
